# Neurophysiological and Autonomic Correlates of Metacognitive Control of and Resistance to Distractors in Ecological Setting: A Pilot Study

**DOI:** 10.3390/s24072171

**Published:** 2024-03-28

**Authors:** Michela Balconi, Carlotta Acconito, Roberta A. Allegretta, Laura Angioletti

**Affiliations:** 1 International Research Center for Cognitive Applied Neuroscience (IrcCAN), Catholic University of the Sacred Heart, Largo Gemelli 1, 20123 Milan, Italy; michela.balconi@unicatt.it (M.B.); robertaantonia.allegretta1@unicatt.it (R.A.A.); laura.angioletti1@unicatt.it (L.A.); 2 Research Unit in Affective and Social Neuroscience, Department of Psychology, Catholic University of the Sacred Heart, Largo Gemelli 1, 20123 Milan, Italy

**Keywords:** resistance to distractors, metacognition, decision-making, EEG, autonomic measures, personality profiles

## Abstract

In organisational contexts, professionals are required to decide dynamically and prioritise unexpected external inputs deriving from multiple sources. In the present study, we applied a multimethodological neuroscientific approach to investigate the ability to resist and control ecological distractors during decision-making and to explore whether a specific behavioural, neurophysiological (i.e., delta, theta, alpha and beta EEG band), or autonomic (i.e., heart rate—HR, and skin conductance response—SCR) pattern is correlated with specific personality profiles, collected with the 10-item Big Five Inventory. Twenty-four participants performed a novel Resistance to Ecological Distractors (RED) task aimed at exploring the ability to resist and control distractors and the level of coherence and awareness of behaviour (metacognition ability), while neurophysiological and autonomic measures were collected. The behavioural results highlighted that effectiveness in performance did not require self-control and metacognition behaviour and that being proficient in metacognition can have an impact on performance. Moreover, it was shown that the ability to resist ecological distractors is related to a specific autonomic profile (HR and SCR decrease) and that the neurophysiological and autonomic activations during task execution correlate with specific personality profiles. The agreeableness profile was negatively correlated with the EEG theta band and positively with the EEG beta band, the conscientiousness profile was negatively correlated with the EEG alpha band, and the extroversion profile was positively correlated with the EEG beta band. Taken together, these findings describe and disentangle the hidden relationship that lies beneath individuals’ decision to inhibit or activate intentionally a specific behaviour, such as responding, or not, to an external stimulus, in ecological conditions.

## 1. Introduction

In organisational contexts, professionals are frequently required to make decisions rapidly and dynamically, interrupting workflows and, when necessary, prioritising unexpected external inputs deriving from multiple sources, a process that can often be effortful, and it is not always successful [1,2].

Indeed, to efficiently manage a decision and, at the same time, be able to correctly select the stimuli that deserve immediate attention and inhibit the behaviour towards distractors, an essential role is played by executive functions (EFs). According to Miller and Cohen, indeed, EFs are high-level cognitive processes that are crucial for managing attentional resources, impulsive actions, and promoting behaviour [3]. Thanks to EFs, an individual is indeed able to inhibit automatic responses and impulses, manipulate and recall information from memory, and respond flexibly to environmental changes [4].

In particular, three partially separable components of EFs are involved in the decision-making process: (i) updating, understood as the constant monitoring and rapid addition and/or deletion of working-memory contents; (ii) shifting, in terms of flexible switching from one task to another or from one mental set to another; and (iii) inhibition, the ability to deliberately override dominant or predominant responses [5,6]. Moreover, according to Friedman et al. (2008), inhibition can be defined as the lowest common denominator in all these functions [7]. Inhibition, in fact, which is responsible for the active maintenance and goal management of the current task, is included in the EFs of updating and shifting, which, however, also involve other abilities. For example, in the updating function, there is not only the deliberate inhibition of certain responses, but also the monitoring of the current contents of working memory. Similarly, in the shifting function, it is necessary to implement a process of inhibiting the less salient information from time to time, to move from one task to another flexibly.

The ability to regulate the inhibition process and the subset of control mechanisms responsible for monitoring cognitive control is called metacontrol [8]. The function of metacontrol consists of adopting different control modalities in response to various tasks, difficulties, or different possible consequences [9], according to a cost–benefit analysis [10,11].

Specifically, according to a review of the scientific literature, there are two distinct control systems—differing in flexibility and computational cost—that reflect two decision-making strategies [12,13]: model-free decision-making and model-based decision-making [12,14]. These decision-making strategies represent a distinction between automatic and deliberative modes of information processing [15].

Model-free decision-making is defined as a reflexive strategy, in which decisions are made based on previously experienced and learned action–reward associations [13]. It is, therefore, a simple strategy, which can be inaccurate and ineffective if people face new stimuli/situations that have not been previously experienced and learned. From this perspective, then, this process can be effective and facilitate decision-making in familiar and well-known situations, but it can also be more energy-consuming and less effective in ambiguous, new contexts and in response to distracting elements that may not have been previously experienced, and to which one is not used to responding. On the other hand, model-based decision-making is defined as a deliberative and prospective strategy, in which decisions are made by evaluating different choice options and their respective consequences. This strategy is usually more accurate and effective, but it also requires greater cognitive effort. At the same time, however, making decisions based on this strategy provides higher behavioural flexibility because dynamic changes in the environment can be accounted for more quickly.

In this theoretical framework, it is important to highlight that cognitive control often requires a balance between goal persistence and flexibility as a key component of decision-making [16]. Goal persistence can help to focus on relevant information and suppress irrelevant external stimuli, which, because of their possible salience, may trigger automatic responses in individuals, distracting them from the cognitive process. In this regard, it can also increase the likelihood of the cognitive system becoming too inflexible and insensitive to alternative possibilities and to important external features of the environment. In turn, adopting a flexible approach facilitates switching between alternative possibilities and actions; however, it can increase the probability of distraction and possible mistakes between cognitive representations [16,17].

The Metacontrol State Model (MSM), a model developed by Hommel, can be used to better understand how this balance works [8]. According to the MSM, decision-making is understood as a process of competition between options based on goals, and an optimal decision-making process can be described as a balance between persistence and flexibility. Thus, if two or more goal-related representations compete for consideration, selection requires one alternative to pass a certain threshold, which, given mutual inhibition, would result in the relative inhibition of the unselected alternative. The individual metacontrol state, which ranges from persistence to flexibility, determines the extent to which alternatives compete and the extent to which they are affected by current goals. Extreme flexibility would consist of minimal competition, while extreme persistence would consist of significant mutual competition [8]. In this sense, cognitive control can be defined not only as a top-down mechanism [18], but also as a bottom-up process triggered by contextual elements, indicating an automatic aspect of cognitive control [19].

In addition, previous studies [20,21] identified two different types of metacontrol: (i) proactive metacontrol, according to which a list of all goal representations is maintained in memory and can be activated at the beginning of any decision-making process, allowing individuals to anticipate and prevent the possible interference of distractors [22], and (ii) reactive or transient metacontrol, in which goal representations are activated and retrieved only after the beginning of a specific decision-making process and following the detection of a specific interference [22].

To sum up, proactive metacontrol allows behaviours to be continuously modified to facilitate the achievement of the goal [23], but it is effortful, given that it requires a high level of consumption of cognitive resources [22,23], while reactive metacontrol is effortless, since it makes it possible to allocate one’s resources to several tasks simultaneously. However, in this last type of metacontrol, there is a greater dependence on the eliciting events themselves because, if they are not sufficiently salient or discriminating, they will not result in the reactivation of the goal [23].

To study metacontrol and the degree to which the “intentional” and “automatic” pathways affect decision-making processes, performance tests and tasks derived from the field of cognitive psychology, such as the Stroop test [24,25] and the Simon effect [26], were widely used in previous works. However, two main limitations characterise such works. First, these tasks do not measure specifically and in an ecological way the ability to resist and control contextual distractors when taking a decision [27], as a proxy of a successful decision-making process in organisational contexts. Secondly, to obtain a more comprehensive and deep study of the phenomenon, it is possible to adopt a multi-methodological approach that combines multiple assessment levels (i.e., the behavioural, neurophysiological, autonomic, and self-report levels) and allows a focus not only on the explicit components of metacontrol (e.g., the behavioural correlates), but also on the implicit components, such as the neurophysiological basis of the process. This multimethodological approach was exploited in previous basic neuroscience research [28], as well as in applied neuroscience studies. The aims were to deepen the influence of and resistance to nudge decision-making in a group of professionals when performing a behavioural task in ecological conditions [29], as well as to explore the neurophysiological correlates of a novel behavioural task assessing decision-making functions [30].

Indeed, the behavioural level, encompassing behavioural measures of performance [such as the participant’s response, the response times (RTs)] and the individual’s metacontrol ability (in terms of consistency and awareness of his/her behavioural performance), can be complemented by the neurophysiological and autonomic level, providing information on the contribution of the central nervous system (CNS) and autonomic nervous system (ANS) to the process.

Starting from the behavioural level, in addition to the participants’ responses, RTs can be considered as indirect workload measures to evaluate participants’ cognitive effort during the performance of a task. For example, collecting RT during a task designed to explore the ability to resist and control contextual distractors may provide insight into the cognitive load and effort required to implement an inhibitory process. In particular, longer RTs reflect greater task-related effort and, in turn, might suggest greater effort in inhibiting automatic behaviours elicited by salient external cues.

The literature suggests various ways to measure an individual’s metacontrol ability in terms of the consistency and awareness of behavioural performance [31]. The most widely used method to assess metacontrol is to directly ask participants to label their experience while performing a task. For example, Wenke et al. explore the sense of control and overall confidence in one’s judgments on a scale ranging from 0 to 100 [32]. Questienne et al. (2018), instead, tested the metacognitive experience of response conflict by asking their participants to answer the question, “*Do you think there was a conflict between the two arrows in this process*?” by choosing from several possible alternatives related to whether the participant believed there was a conflict [33].

Secondly, regarding the CNS markers, electrophysiological (EEG) frequency bands (i.e., delta, theta, alpha, beta, and gamma), their functional meaning and their brain localisation make it possible to understand the cognitive and attentional effort in the information processing [34] and emotional processing [28] associated with decision-making [35] and metacontrol. For instance, Basharpoor et al. (2021) demonstrated a relationship between EFs and the EEG activity of the theta, beta, and alpha bands in the frontal regions; specifically, frontal brain activity is associated with EFs and cognitive control [36]. Furthermore, the theta band has been associated with an increased cognitive control mechanism in people who are less prone to taking risks and changing their decision-making process [37,38]. The beta band, instead, can be interpreted as an index of active attention and engagement [34]. The activation of this specific band might also be associated with inhibitory control, which, as mentioned above, could be an essential skill for resisting and controlling contextual distractors in the decision-making process [39]. On the other hand, a greater activation of the alpha band is an indicator of cognitive effort and engagement during the decision-making process, as shown by Runnova et al. [40]. Additionally, the presence of the alpha band in the parietal areas has been interpreted as an index of attentional requests from the environment [41].

Thirdly, focusing on ANS measures, the electrodermal activity (EDA) and cardiac parameters provide information on the ANS responsiveness in terms of electrodermal (skin conductance Level—SCL, skin conductance response—SCR) or cardiac activity (i.e., heart rate—HR, heart rate variability—HRV) through an autonomic measurement recording tool (e.g., biofeedback) [42]. In particular, within the research conducted on decision-making processes, Dawson et al. (2011) highlighted how the SCR reflects the anticipation of a potential negative outcome and its absence might instead be associated with the decision not to take any risks during the decision-making process [43]. Regarding the inhibitory process, a decrease in HR has been shown to be related to intentional action and inhibition [44], which is more pronounced in complex situations [45], such as the decision-making process. Furthermore, ANS indices have been identified as correlates of emotional processes, such as emotional engagement or stress levels, during moral-decision-making tasks [46,47].

Fourthly, concerning the self-report level, it should be noted that different personality profiles have also been associated with the manifestation of specific EEG frequency bands. For example, Li et al. found associations between the alpha and beta bands over the frontal area and the agreeableness personality profile, and between the theta and beta activities in the temporal and parietal area and the conscientiousness personality. Furthermore, the extraversion profile was shown to correlate with the delta and beta bands in the frontal and with the theta in the occipital areas [48].

Moreover, there is considerable evidence that personality is associated with human decision-making performance: in particular, it has been demonstrated that an impulsive personality is linked to risky decisions in the social, ethical, and gambling domains, whereas anxiety is linked to risk-averse choices [49]. Additionally, the personality attribute of extraversion is connected to the inhibitory process [50] and is found to be central to metacognitive awareness, as well as the personality profile of openness [51]. Finally, a recent finding suggests the association between a high level of conscientiousness and agreeableness and the inhibition EF [52].

Therefore, when adopting a multimethodological approach, it proves interesting to include self-report measures of individuals’ characteristics, such as the 10-item Big Five Inventory (10-item BFI) [53], which allows the highlighting of five specific personality profiles: extroversion, agreeableness, conscientiousness, emotional stability, and open-mindedness. It is also pertinent to suppose that different personality profiles might be associated with different levels of ability to resist distractors, as well as with the activation of specific neurophysiological and autonomic patterns.

Within this theoretical framework, this study applied a multimethodological neuroscientific approach to investigate the behavioural, neurophysiological, autonomic, and self-report correlates of the ability to resist and control ecological distractors during an ecological-decision-making task. The participants performed a novel Resistance to Ecological Distractors (RED) task while neurophysiological and autonomic data were continuously detected through the recording of EEG and autonomic measures. At the end of the task, the 10-item BFI [53] was administered to investigate the individuals’ personality profiles.

The goal of this research was to explore whether a specific behavioural, neurophysiological, autonomic pattern activated during the task is correlated with specific personality profiles.

Specifically, with respect to behavioural data, it was first hypothesised that a correlation would be identified between the participants’ ability to resist and control contextual distractors (computed in an index considering each participant’s behavioural responses and RTs) and the coherence and awareness of their behaviour (i.e., metacognitive ability). Specifically, it was expected that the increased ability to resist and control distractors, involving the adoption of a persistence approach, would lead the participants to becoming more aware of what had been done.

With reference to the relationship between behavioural indices of resistance, metacognition and neurophysiological (EEG frequency bands), we expected to observe a positive correlation between the theta band power in the frontal brain regions, as a marker of increased cognitive control [37,38], and the ability to resist and control contextual distractor stimulus and metacognition. In addition, we expected to find a positive correlation between the power of the beta band in the frontal areas, as an indicator of attention and involvement [34], and the same behavioural correlates (the ability to resist ecological distractors and metacognition).

Concerning the ANS indices, it is thought that the inability to resist and control contextual distractor stimulus could be associated with higher levels of SCR and HR, as an index of emotional and cognitive engagement [46,47]. Indeed, it is possible to suppose that a consideration of and elaboration on the distractor stimulus could involve a more flexible approach and since, with this approach, the individual switches between different goals, greater cognitive effort and emotional engagement are required, as the main information-processing process is interrupted to respond to the external stimulus and resumed later [16,23]. On the other hand, a decrease in HR may be correlated with the ability to resist and control distractors, as an index of the inhibition process [45].

Finally, with reference to the relationship between a specific neurophysiological profile supporting task performance and personality profiles, it can be supposed that a lower level of agreeableness is associated with greater activation in low-frequency bands (i.e., theta) during task performance, as an indication of an increased cognitive control mechanism in people who are less prone to taking risks and to changing their decision-making processes [37,38]. Similarly, a higher level of agreeableness and extroversion could be associated with higher task-related activity in the beta band, as an index of active attention and engagement [34]. Conscientious people, on the other hand, may have less activation of the alpha band, as an indicator of cognitive effort and engagement during the decision-making process [40] in individuals who tend towards precision, accuracy, and success.

Furthermore, regarding the relationship between the autonomic indices supporting task performance and personality profiles, a higher level of extroversion would be expected to correlate with a decrease in HR while performing a task, as an extroverted personality is focused, engaged, and concentrated on gathering as much detail and stimuli as possible about the task it is performing, inhibiting possible external influences [54].

## 2. Materials and Methods

### 2.1. Sample

In total, 24 healthy individuals, 11 females and 13 males, with an average age of 35.33 years (Standard Deviation_age_ = 11.70) were recruited to participate in this study voluntarily and without receiving any compensation for their participation. The sample was defined according to the following exclusion criteria: history of neurological or psychiatric disorders, severe depressive episodes, high level of stress, low global cognitive functioning, and undertaking therapy based on psychoactive drugs that can alter cognitive decision-making functioning.

In addition, all individuals had normal-to-corrected vision and, to be included in the experimental sample, they signed their written informed consent. Moreover, the sample members were right-handed and there were no differences in education level or type of profession. The experimental study was conducted in accordance with the Helsinki Declaration (2013) and approved by the Ethics Committee of the Department of Psychology, Catholic University of the Sacred Heart of Milan in Italy.

### 2.2. Procedure

The whole experimental procedure lasted approximately 20 min and was conducted in a dedicated and quiet room where participants sat in a comfortable chair in front of a computer monitor 80 cm from their faces. After being fully instructed on the experimental procedure, the enrolled individuals filled in their informed consent. Subsequently, the EEG wearable MUSE^TM^ headband (version 2; InteraXon Inc., Toronto, ON, Canada) was applied to the participants’ heads and the X-pert2000 portable Biofeedback (Schuhfried GmbH, Modling, Austria) was placed on the non-dominant hand for recording a total of 120 s of neurophysiological and autonomic resting-state baseline. Participants were then presented with the RED task and their neurophysiological and autonomic activity was collected continuously through the task.

**Figure 1:**
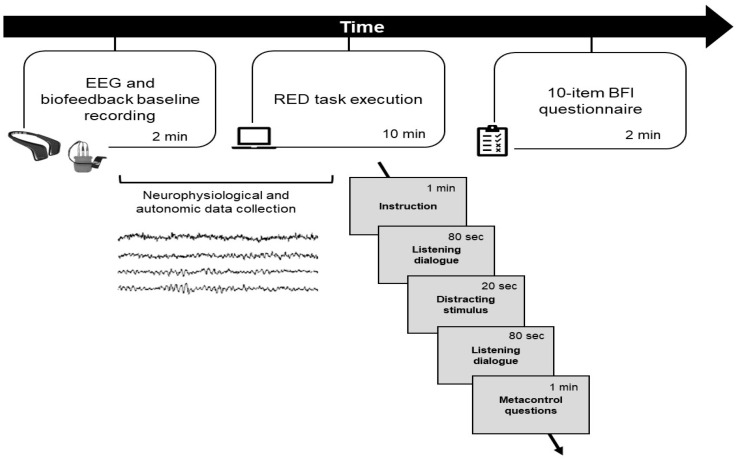
Experimental procedure. The figure shows the experimental procedure with the EEG wearable MUSE^TM^ headband and the X-pert2000 portable Biofeedback adopted to collect EEG and autonomic activity during the duration of the RED task. At the end of the task, the 10-item Big Five Inventory questionnaire was administered.

Following task completion, participants filled in the 10-item Big Five Inventory questionnaire [53]. Figure 1 shows the experimental setup with the EEG wearable MUSE^TM^ headband, the X-pert2000 portable Biofeedback, and the computerised task.

#### 2.2.1. The Resistance to Ecological Distractors Task (RED Task)

The Resistance to Ecological Distractors (RED) task was administered via a web-based experiment-management platform (PsyToolkit, version 3.4.4) [55,56] and was designed to assess individuals’ ability to optimally resist and control distractors ecologically defined by contextual cues in decision-making conditions. The participants were presented with two realistic decision-making scenarios, in which they were asked to identify themselves and make decisions.

Each scenario consisted of listening to a dialogue between two persons. The participant was asked to understand the dialogue and count the number of times a specific background sound was presented during the dialogue. In the middle of the dialogue, participants were presented with a distracting stimulus: in particular, on the screen appeared the notification of a call coming from a colleague. The participant had to decide whether to accept or reject the call. After this decision, the dialogue resumed and concluded (see Table 1 for the scenarios).

For instance, in the first scenario, participants received the following instructions and reminder:

*“Today is a particularly busy working day. You ate fast in the cafeteria aware that at 2:00 p.m. you have one last update meeting for a project that needs to be finished within two weeks. Attention: during the meeting, you will listen to, you will have to understand the dialogue and count how many times you hear a sound similar to a notification”.*

*“Reminder: Recall as soon as possible Roberto Rossi (project manager of the company).”*

At the end of each dialogue, the participants were explicitly asked to (i) indicate their decision regarding the distractor stimulus (whether they accepted or rejected the call), (ii) justify their decision by selecting a specific reason for their choice among various answer options, and (iii) specify the number of times they heard the background sound.

On the basis of their decision regarding the distractor stimulus (whether they accepted or rejected the call), they were presented with one of the following multiple-choice questions.

If participants chose to respond to the colleague’s call that appeared during the dialogue, they had to justify that choice by selecting one of the following options:

(a) *No doubt it was more important to respond immediately to the call because there was a greater need to speak with the colleague as soon as possible;*
(b) *It seemed better to me to solve first the question of the pending call and then dedicate myself to the meeting;*
(c) *Since the reminder had been set at that time, it was important to respect the commitment made;*
(d) *I reacted spontaneously, without thinking too much about it;*
(e) *I did not make a choice.*

Conversely, if the participants decided to decline their colleagues’ call, they had to justify the choice made by selecting one option from the following alternatives:

(a) *No doubt it was more important to follow the meeting so as not to lose track of the sounds;*
(b) *If I had answered, I would have lost important information about the project, which needs to be finished soon;*
(c) *It seemed better to do one thing at a time;*
(d) *I reacted spontaneously, without thinking too much about it;*
(e) *I did not make a choice.*

This last question was administered under time pressure, with 20 s as the maximum time window to provide a response.

#### 2.2.2. Behavioural Data Acquisition

For the behavioural data, both response scores (whether they accepted or rejected distractor stimulus, [i.e., the call] and the option selected to justify the choice) and RTs were collected for each type of scenario and then transcribed offline to create two behavioural indices, the Resistance Index (Res-i) and the Metacognition Index (Met-i), respectively.

The Res-i was calculated through the following steps. First, a score of four points was assigned if the participant did not respond to the distractor stimulus in either scenarios, two points if the participant responded in only one scenario, and zero points if the participant responded in both scenarios. This score was then converted into a common decimal metric scale and composed the Res-i, an index measuring whether an individual is able to resist and control distractors ecologically defined by contextual cues in the decision-making process.

The Met-i was computed through the following steps. First, a score ranging from one to five points was assigned on the basis of the type of option chosen to justify the decision made on the multiple-choice questions. In this case, five points were given if the chosen justification reflected a high level of consistency in the actual behavioural performance. For instance, if the participant chose to respond to the colleague’s call that appeared during the dialogue and selected option (a), “*No doubt it was more important to respond immediately to the call because there was a greater need to speak with the colleague as soon as possible*”, he/she obtained five points. Similarly, if the participant decided to decline their colleague’s call and selected option (a), “No doubt it was more important to follow the meeting so as not to lose track of the sounds”, he/she obtained five points. For both multiple-choice questions, fewer points were attributed if the chosen alternative represented a lack of reflection on the participant’s behaviour, which then occurred almost spontaneously (e.g., two points if the participant selected option (d), “*I reacted spontaneously, without thinking too much about it*”. In both cases, if participants selected option (e), “*I did not make a choice*”, they obtained only one point.

The scores derived from the two scenarios were then averaged and converted to a decile scale.

The RTs were collected for the answers from the two scenarios, divided by the total allowed time (20 s), averaged, and converted in a decile scale.

These scores were then used to calculate Met-I, which is a ratio between the response score and the RTs, both expressed in decile scale. The Met-i measures the level of consistency between the decision made and the justification given in the management of distractors.

#### 2.2.3. Big Five Inventory: Self-Report Data Acquisition

The Italian version of the 10 item-BFI [53] was administered to collect information on individuals’ personality dimensions. Through a total of 10 items, this inventory makes it possible to measure five different dimensions of personality: (i) extroversion; (ii) agreeableness; (iii) conscientiousness; (iv) emotional stability; and (v) open-mindedness. The scale consists of an initial statement, “I see myself as a person who…”, and the participant is asked to respond to each statement that describes his/her personality. Participants are asked to respond on a five-point Likert-type scale ranging from 1 (“strongly disagree”) to 5 (“strongly agree”). Higher mean scores on a subscale indicate a greater presence of that specific personality profile.

#### 2.2.4. The Muse^TM^ Headband for Neurophysiological Data Acquisition

The Muse^TM^ headband (version 2; InteraXon Inc., Toronto, ON, Canada) was employed to collect, in a non-invasive manner, the neurophysiological data and to measure EEG spectral activity changes, between the resting-state baseline and task phases. This wearable tool, indeed, permits the detection of EEG neurophysiological activity via an accelerometer, gyroscope, pulse oximetry, and seven electrodes. Of these seven electrodes, specifically, three are used as references, while the others detect the EEG spectral activity in the frontal (AF7 and AF8, left and right forehead, respectively) and temporoparietal (TP9 and TP10, left and right hemisphere, respectively) areas, according to the international 10–20 system [57]. These electrodes were made of conductive material (silver) and silicon rubber.

The data were gathered at a sampling rate of 256 Hz via the mobile app Mind Monitor and transferred via Bluetooth to the associated smartphone. Participants were instructed to minimise their movements, in order to reduce artifacts in the EEG signal. Mind Monitor applied a 50-hertz notch frequency filter, and data were then visually inspected to remove motor artifacts (such as jaw clenching and eyeblinks). Through the logarithm of the power spectral density of the raw EEG data from each channel, raw data were transformed by using a Fast Fourier Transform (FFT) into brain waves at various frequency bands: delta (1–4 Hz), theta (4–8 Hz), alpha (8–13 Hz), beta (13–30 Hz), and gamma (30–44 Hz). The recording of a 120-s baseline took place at the beginning of the experimental phase and, for each participant, EEG activity during the experimental conditions was weighted over baseline values.

#### 2.2.5. The X-Pert2000 Biofeedback for Autonomic Data Acquisition

The X-pert2000 portable Biofeedback system with a MULTI radio module (Schuhfried GmbH, Modling, Austria) was employed to collect, in a non-invasive manner, the autonomic data and to identify possible variations in skin conductance and cardiovascular parameters, between the resting-state baseline and task phases. This portable tool, indeed, permits the detection of peripheral parameters, such as skin conductance level (SCL), skin conductance response (SCR), heart rate (HR), and heart rate variability (HRV), via a sensor placed on the distal phalanx of the second finger of the non-dominant hand.

The data for skin conductance parameters (SCL and SCR) were gathered with an electrodermal activity (EDA) gold electrode. On the other hand, the data on cardiovascular parameters (HR and HRV), measured in beats per minute (bpm), were gathered with photoplethysmography. To prevent non-dominant-hand movements from interfering with the recordings, the accelerometer of the transmission unit was used, calibrated in meters/second squared (m/s^2^).

### 2.3. Data Analyses

To explore whether a specific behavioural, neurophysiological, or autonomic pattern activated during the task was correlated with specific personality profiles, the following steps in analysis were performed. Before the analyses, the normality of data was tested with Shapiro–Wilk and the normality of the data was confirmed.

First, a Pearson correlation between the two behavioural indices (Res-i and Meta-i) was performed, to explore a potential relationship between task performance, in terms of the ability to resist ecological and contextual distractors, and metacognition, understood as the level of consistency between the decision made and the motivation given in the management of distractors.

Secondly, the two behavioural indices (Res-i and Meta-i) were separately correlated with EEG frequency bands (delta, theta, alpha, beta, and gamma) for the four electrodes (AF7, AF8, TP9, TP10) and, secondly, with the autonomic indices (SCL, SCR, HR, HRV) recorded during task performance. This step in analysis was performed to explore whether a specific neurophysiological or autonomic profile supported task execution or the metacognitive phase.

Finally, to check whether the neurophysiological or autonomic profile identified during the task execution or the metacognitive phase was related to specific personality profiles, Pearson correlations were performed between each of the EEG frequency bands (delta, theta, alpha, beta, gamma) for the four electrodes (AF7, AF8, TP9, and TP10) recorded during task performance and the 10-item BFI subscales scores, as well as between the autonomic indices (SCL, SCR, HR, HRV) recorded during task performance and the 10-item BFI subscales scores.

## 3. Results

**Figure 2:**
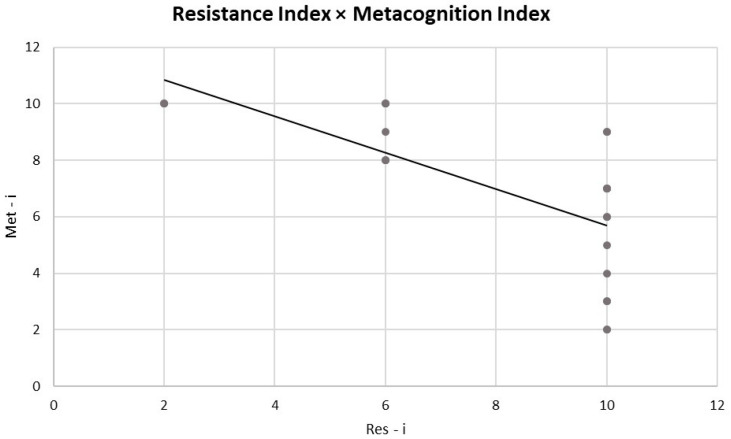
Pearson correlations between behavioural indices. The scatter plot displays a significant negative correlation between Res-i and Meta-i. The straight line represents the global linear trends.

For the first step in the analysis, the Pearson correlation performed between Res-i and Meta-i showed a significant negative correlation (r = −0.694, *p* ≤ 0.001) (Figure 2).

**Figure 3:**
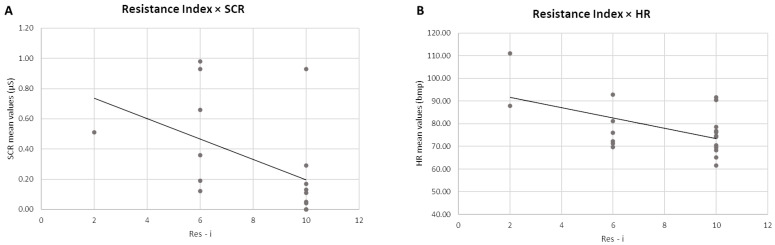
Pearson correlations between behavioural and autonomic indices. (**A**) The scatter plot displays a significant negative correlation between Res-i and mean SCR values. (**B**) The scatter plot displays a significant negative correlation between Res-i and mean HR values. The straight lines represent the global linear trends.

In the second step in the analysis, negative correlations were found between the Res-i and the mean SCR values (r = −0.483, *p* = 0.050) and between the Res-i and mean HR values (r = −0.533, *p* = 0.016) (Figure 3A,B). No other significant correlations were found between the Res-i and the autonomic data, nor for the Meta-i. No significant correlations were found between the behavioural indices (Res-i and Meta-i) and the EEG data.

**Figure 4:**
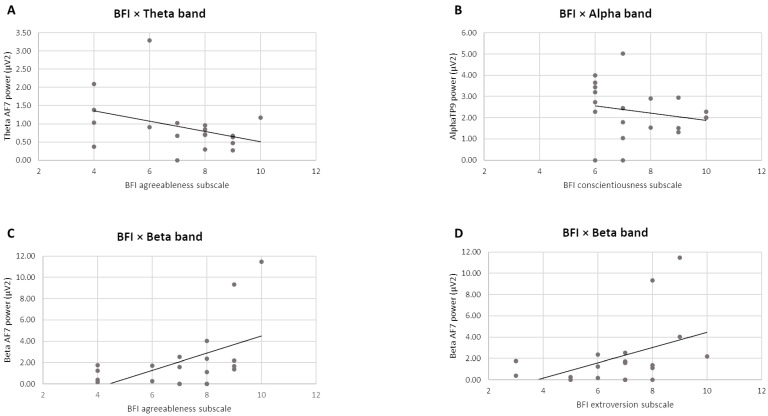
Pearson correlations between 10-item BFI scores and EEG indices. (**A**) The scatter plot displays a significant negative correlation between theta band power and agreeableness profile. (**B**) The scatter plot displays a significant negative correlation between alpha band power and conscientiousness profile. (**C**) The scatter plot displays a significant negative correlation between beta band power and agreeableness profile. (**D**) The scatter plot displays a significant positive correlation between beta band power and extroversion profile. The straight lines represent the global linear trends.

Concerning the third step in the analysis, Pearson correlations between the 10-item BFI subscales and the four electrodes of the EEG frequency bands’ power showed significant correlations for the theta, alpha, and beta bands. No significant correlations were found between the gamma and delta bands. Specifically, for the theta band, a negative correlation was found between the 10-item BFI subscale of agreeableness and the theta band in AF7 (r = −0.489, *p* = 0.040) (Figure 4A). A negative correlation was also reported between the 10-item BFI subscale of conscientiousness and the alpha band in TP9 (r = −0.495, *p* = 0.043) (Figure 4B). Finally, the beta band power in AF7 was correlated positively with the 10-item BFI subscale of agreeableness (r = 0.572, *p* = 0.021) and the 10-item BFI subscale of extroversion (r = 0.509, *p* = 0.044) (Figure 4C,D).

**Figure 5:**
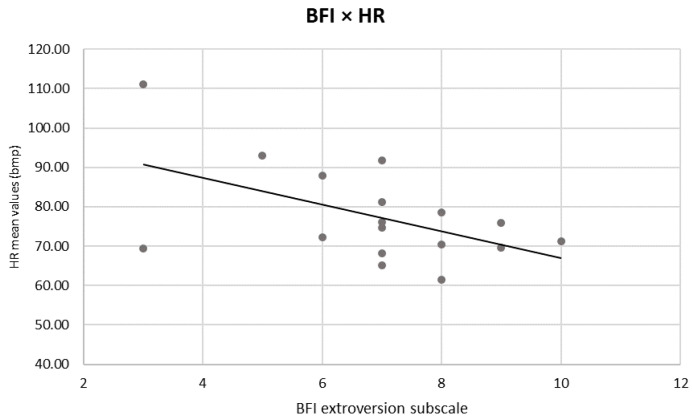
Pearson correlations between 10-item BFI scores and autonomic indices. The scatter plot displays a significant negative correlation between HR and extroversion profile. The straight line represents the global linear trends.

Lastly, a negative correlation was found between the 10-item BFI subscale of extroversion and the mean HR values (r = −0.526, *p* = 0.030) (Figure 5).

## 4. Discussion

By adopting a multimethodological neuroscientific approach, this study explores whether a specific behavioural, neurophysiological, and autonomic pattern activated during an ecological decision-making task (the RED task) is correlated with specific personality profiles in healthy individuals. In this work, the RED task was designed to assess individuals’ ability to resist and control ecological distractors defined by contextual cues in decision-making conditions and to measure the coherence and awareness of individuals’ decisions (i.e., metacognitive ability). The results obtained by analysing the relation between the behavioural, neurophysiological, autonomic, and self-report data during the task are discussed below.

First, regarding the behavioural level of this multimethodological approach, a negative correlation was found between the Res-i and Meta-i indices in relation to task execution and the metacognitive phase. This result can be interpreted by considering two perspectives.

According to the first perspective, this significant negative correlation could suggest that the greater ability to resist and control ecological distractors is related to a lower level of awareness of these distractors. Indeed, it might be plausible that the more a person is effective in his/her performance (and is able to resist distractors), the less awareness is necessary for them to have self-control over their own behaviour: in other words, the person has automated their resistance behaviour effectively and, thus, self-control in the performance is no longer necessary.

If we read this result from another perspective, a lower ability to resist and control ecological distractors is related to a higher level of awareness of these distractors. Even this behavioural situation can be plausible. In fact, it can be argued that displaying higher levels of awareness and self-control over behaviour while executing a task under pressure can negatively affect behavioural performance. Monitoring a behaviour that should be automated and self-monitoring during performance can influence RTs and lead to distraction from one’s performance.

Both these perspectives feature strengths and weaknesses and, perhaps, adopting a balanced or flexible approach may represent the best attitude. This balance might be even more important and valuable when considering the organisational setting, where professionals are often required to make decisions quickly and dynamically, in some cases prioritising unexpected external inputs [1,2]. Indeed, it would be desirable for an individual (and especially a professional) to be able to isolate himself/herself to ensure maximum concentration on a given task, but also for the individual to always be ready to seize salient and useful external stimuli to effectively complete decision-making processes [58]. From this perspective, future work could consider replicating this study by adopting a between-subject approach, perhaps including a sample of managers, to explore potential group differences in terms of professional background, age, or expertise. In fact, it has been shown that achieving a balance between persistence and flexibility may depend not only on the type of task, but also on the individual’s age [59].

Secondly, correlational analyses were performed to explore whether a specific neurophysiological or autonomic profile supports task execution or the metacognitive phase. Concerning the link between the behavioural indices and the CNS markers, no direct correlations were found between the behavioural indices and the EEG data. This is because this report lacked the consideration of specific personality profiles as mediators of this relationship. Indeed, as demonstrated by the significant correlation results described below, specific personality profiles were correlated with the EEG data during task performance, and they could play a key role in mediating the decision-making process.

On the other hand, the analysis performed on the relationship between the behavioural indices and ANS indices showed two significant results: (i) a negative correlation between Res-i and the mean SCR values, and (ii) a negative correlation between Res-i and the mean HRs values during task performance. Focusing on the relation between the decrease in SCR index and the increased ability to resist ecological distractors, it is possible to explain this result by considering that the goal-persistence approach [16] and the consequent inhibition of automatic behaviours elicited by salient external stimuli may be due to a desire to avoid taking risks during decision-making. An SCR response is indeed definable as an index of the anticipation of significantly adverse outcomes related to decision-making [43,60].

Furthermore, the decrease in HR during the task in relation to the ability to resist and control ecological distractors can be interpreted as a marker of the inhibitory process put in place towards external stimuli to enable one to keep focused on a goal [44]. On the other hand, if we consider the increase in both these ANS indices in relation to the inability to resist ecological distractors, they can be interpreted as markers of emotional involvement, greater effort, and an increase in stress levels [46], which may occur as a result of adopting a flexible approach that allows switching between possibilities and alternative actions [15].

Finally, some interesting results were found thanks to the analysis performed to check whether the neurophysiological or autonomic activity identified during the task execution or the metacognitive phase was related to specific personality profiles.

Significant correlations were found between the theta, alpha, and beta bands activated during the task and some personality profiles measured with the subscale of the 10-item BFI.

The negative correlation between the subscale of agreeableness and the theta band in the left frontal regions (AF7) can be explained by focusing on the characteristics of this personality profile. Indeed, a person with high levels of agreeableness is characterised by traits such as cooperativeness and empathy, and has a predisposition toward external aspects [61]. Thus, individuals with high agreeableness, who pay attention to external stimuli, might show a lower presence of low-frequency bands, such as the theta band, in frontal brain sites, since they activate cognitive and inhibitory control mechanisms less, which are instead typically characterised by a high presence of the theta band in the frontal areas [37,38].

In addition, the negative correlation between the conscientiousness subscale and the alpha band in the left temporo-parietal regions (TP9) is in line with the existing literature. In fact, according to Runnova et al. (2021), reduced activation of the alpha band is to be interpreted as an index of cognitive effort and commitment during the decision-making process [40], which is definitely higher in individuals who tend towards precision, accuracy, and success, such as people characterised by the conscientiousness profile [62].

Moreover, both the agreeableness and the extroversion subscale were positively correlated with beta band presence in the left frontal area (AF7). The association between beta band power and both agreeableness and extroversion reflect the prediction models developed by Li et al. [48]. In fact, according to the researchers, for the agreeableness model, the largest contribution was observed in the beta band activation in the frontal area, among others, and, similarly, a frontal distribution of the beta band was also found among the profiles in the extroversion models. Additionally, since greater beta band activation is predictive of active attention and engagement [34], it is likely that cooperative, empathic people who are inclined towards external stimuli, such as those characterised by the profile of agreeableness [61], as well as those who are dynamic, those who seeking emotions, and focused, as extroverts [62], are more involved and engaged in tasks [63,64].

The presence of positive correlations between specific personality profiles and EEG parameters exclusively in the left hemisphere (AF7 and TP9) can also be explained by focusing on the characteristics of the sample, which, as described above, consisted of right-handed people.

Lastly, a negative correlation was found between the subscale of extroversion and the mean HR values. As mentioned above, a person with an extroverted personality [62] can be focused and engaged and able to concentrate on an external goal (e.g., the task that is performed) [63,64] and can tend to inhibit possible external distractors [54]: this inhibitory process can also be characterised by a decrease in HR during task performance [44]. However, the relationship between personality profiles, EEG, and autonomic profiles that characterise the RED task must be explored in depth and, possibly, confirmed by other studies.

## 5. Conclusions

To conclude, this study exploited a newly designed ecological task to demonstrate a relationship between the behavioural ability to resist and control ecological distractors and metacognition. Moreover, it was shown that the ability to resist ecological distractors is related to a specific autonomic profile and how the neurophysiological and autonomic activations that occur during task execution correlate with specific personality profiles. Taken together, these findings are presented in order to describe and disentangle the hidden relationship behind individuals’ decision inhibit or activate a specific behaviour (such as responding, or not, to an external stimulus), consciously or unconsciously, under ecological conditions.

Despite the added value of this work in the study of the ability to resist ecological distractors and metacontrol, some limitations should be considered. Future research should increase the sample size to improve the representativeness and reliability of the current results. Furthermore, it would be interesting to compare the current sample with a sample of professional managers, to explore potential differences in the ability to control and resist ecological distractors in relation to expertise or professional background. Future research may also include a long-term study to determine whether a person’s inhibitory ability to resist ecological distractors can alter over time and in response to environmental circumstances, or whether it can be trained by specific neurocognitive interventions.

Finally, focusing on the multimethodological approach, it might be desirable to develop studies that include other self-report measures, like the General Decision-Making Style [65,66], to profile individuals’ decision-making styles, or additional neuroscientific tools, such as functional near-infrared spectroscopy (fNIRS), to deepen our understanding of whether the hemodynamic variations in specific brain areas can be associated with the ability to resist ecological distractors and metacontrol. Furthermore, future studies may consider the use of statistical tests that determine a cause–effect link (rather than simply a correlational link, as in this study) to infer causality or directionality in the observed relationships between personality traits, neurophysiological responses, and decision-making performance.

Overall, it is important to emphasise that decision-making is one of the most complex high-order processes, which takes into account several variables and can be characterised by unpredictability. However, this study attempted to grasp the complexity of the correlates of the decision-making process by creating an ecologically valid decision-making task that represents a real-life situation.

## Figures and Tables

**Table 1:** The realistic decision-making scenarios with related distractor stimulus and metacognition questions.

Scenario	Distractor Stimulus	Metacognition Questions
Today is a particularly busy working day. You ate quickly in the cafeteria, aware that at 2:00 p.m. you have one last update meeting for a project that needs to be finished within two weeks. Attention: during the meeting you will listen to, you will have to understand the dialogue and count how many times you hear a sound similar to a notification. Reminder: Recall as soon as possible Roberto Rossi (project manager of the company).	Notification of a call coming from a colleague	*Acceptance of the distractor stimulus*
		(a) No doubt it was more important to respond immediately to the call because there was a greater need to speak with the colleague as soon as possible (b) It seemed better to me to solve first the question on the pending call and then dedicate myself to the meeting (c) Since the reminder had been set at that time, it was important to respect the commitment made (d) I reacted spontaneously, without thinking too much about it (e) I did not make a choice
		*Resistance to the distractor stimulus*
		(a) No doubt it was more important to follow the meeting so as not to lose track of the sounds (b) If I had answered, I would have lost important information about the project, which needs to be finished soon (c) It seemed better to do one thing at a time (d) I reacted spontaneously, without thinking too much about it (e) I did not make a choice
As in every year, by the end of this year, you must complete 30 h of training through online courses. You are completing a training session, and you are required to listen to the audio presented carefully, since you will be asked specific questions at the end. If you fail to answer these questions, you will have to start the session over again. Attention: during the training, you will listen to audio recordings, and you will be tasked with understanding the dialogue and counting how many times the word “training” appears. Reminder: You are waiting for an important email about the approval of an investment project; the deadline for submitting this project to your supervisor is today, so it is important to respond to this email as soon as possible after you receive it.	Notification of the e-mail about the approval of the investment project	*Acceptance of the distractor stimulus*
		(a) No doubt it was more important to respond immediately to the e-mail given the close deadline (b) It seemed better to me to solve first the question regarding the pending project’s approval (c) Since my stakeholders were expecting my response within a day, I responded immediately to respect the commitment made (d) I reacted spontaneously, without thinking too much about it (e) I did not make a choice
		*Resistance to the distractor stimulus*
		(a) No doubt it was more important to follow the audio carefully so that I would not have to repeat the training session later if I got something wrong on the test (b) If I had answered, I would have lost important information about the training course (c) It seemed better to do one thing at a time (d) I reacted spontaneously, without thinking too much about it (e) I did not make a choice

## Data Availability

The datasets generated and analysed during the current study are available from the corresponding author upon reasonable request.
